# Tracking the leavers: towards a better understanding of doctor migration from Ireland to Australia 2008–2018

**DOI:** 10.1186/s12960-019-0365-5

**Published:** 2019-05-28

**Authors:** Niamh Humphries, John Connell, Joel Negin, James Buchan

**Affiliations:** 1 Research Department, Royal College of Physicians of Ireland, Dublin, Ireland; 2 School of Geosciences, University of Sydney, Sydney, Australia; 3 School of Public Health, University of Sydney, Sydney, Australia; 4 Queen Margaret University, Edinburgh, UK

## Abstract

**Background:**

The recession of 2008 triggered large-scale emigration from Ireland. Australia emerged as a popular destination for Irish emigrants and for Irish-trained doctors. This paper illustrates the impact that such an external shock can have on the medical workforce and demonstrates how cross-national data sharing can assist the source country to better understand doctor emigration trends.

**Method:**

This study draws on Australian immigration, registration and census data to highlight doctor migration flows from Ireland to Australia, 2008–2018.

**Findings:**

General population migration from Ireland to Australia increased following the 2008 recession, peaked between 2011 and 2013 before returning to pre-2008 levels by 2014, in line with the general economic recovery in Ireland. Doctor emigration from Ireland to Australia did not follow the same pattern, but rather increased in 2008 and increased year on year since 2014. In 2018, 326 Irish doctors obtained working visas for Australia. That doctor migration is out of sync with general economic conditions in Ireland and with wider migration patterns indicates that it is influenced by factors other than evolving economic conditions in Ireland, perhaps factors relating to the health system.

**Discussion:**

Doctor emigration from Ireland to Australia has not decreased in line with improved economic conditions in Ireland, indicating that other factors are driving and sustaining doctor emigration. This paper considers some of these factors. Largescale doctor emigration has significant implications for the Irish health system; representing a brain drain of talent, generating a need for replacement migration and a high dependence on internationally trained doctors. This paper illustrates how source countries, such as Ireland, can use destination country data to inform an evidence-based policy response to doctor emigration.

## Background

Emigration has been a feature of Irish life for centuries [1]. Ireland’s economic downturns of the 1950s and 1980s were accompanied by waves of mass emigration, primarily to England and to the United States of America [2], but Australia has also been a very significant destination [3]. During Ireland’s economic boom, 1995–2008, the then Taoiseach (Prime Minister), Bertie Ahern, claimed to have strengthened the Irish economy and to have ‘delivered… an end to the days of forced emigration’ [2, 4]. However, the collapse of the Irish banking system later that year (2008) and the 2010 bailout by the European Central Bank [5] triggered another wave of large-scale emigration from Ireland.

Much like earlier waves of emigrants, those who emigrated from Ireland post-2008, did so to escape recession and unemployment (which increased from 4 to 14% in 4 years [5]). The collapse of the housing market also served as a driver of emigration—the proportion of residential mortgages in arrears increased from 3% in 2009 to 13% in 2013 [6]. Overall, the number of people emigrating from Ireland increased from 45 000 in 2008 to 87 000 in 2012 [7] although this figure includes EU and non-EU citizens emigrating from Ireland alongside Irish citizens [7]. Skilled workers, including health workers, were a significant part of that emigration.

### Irish emigration to Australia post-2008

**Fig. 1:**
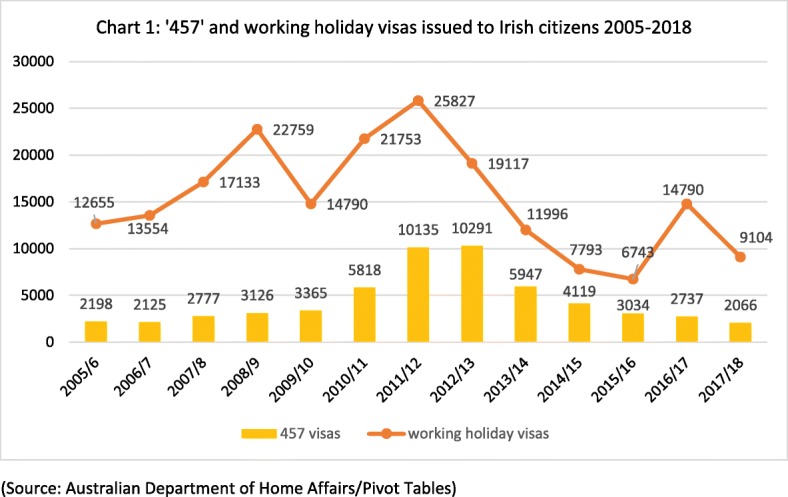
‘457’ and working holiday visas issued to Irish citizens 2005–2018 (source: Australian Department of Home Affairs/Pivot Tables)

As an English-speaking destination country which escaped the worst effects of the global financial crisis more effectively than other countries, Australia again emerged as an attractive destination country for Irish emigrants, post-2008. Emigration data illustrate this popularity. In 2012, 87 000 people emigrated from Ireland, 46 500 of whom were Irish citizens [7]. In the same year, 10 135 Irish citizens were granted ‘457’ working visas and a further 25 827 were granted Australian working holiday visas (see Fig. 1). If all of those granted visas for Australia followed through with their migration plans, that would mean that 77% (35 962/46 500) of the Irish citizens who emigrated from Ireland during 2012, migrated to Australia. This indicates the significance of Australia as a destination for Ireland’s post-2008 emigrants. Although most Irish emigrants to Australia, post-2008, entered Australia on temporary working visas, such as the ‘457’ and working holiday scheme, this does not mean that their migration to Australia was temporary. Pathways to permanent residence and/or citizenship in Australia are available, particularly to skilled migrants.

### Doctor emigration from Ireland post-2008

The emigration of doctors^1^ from Ireland has been an issue for the Irish health system since the onset of recession in 2008/2009. This emigration trend and its impact on the Irish health system is of interest for several reasons:

1. A high rate of doctor emigration represents a loss of capacity in the Irish health system [8]. It also represents a drain of talent and skills from the Irish health system.
2. The emigration of doctors is eroding return on recent state investment in basic medical training in Ireland.
3. Replacement migration has been considerable, as emigrant doctors are replaced in the Irish health system with internationally trained doctors. Ireland’s dependence on internationally trained doctors (measured as a proportion of those registered to practice medicine in Ireland) has increased—from 13.4% in 2000 to 33.4% in 2010 [9], to 37.9% in 2015 [10], to 42% in 2017 [11]. The overall medical workforce^2^ increased by 29% between 2007 and 2017, from 7115 to 9193 [12].
4. A high rate of doctor emigration has coincided with vacancy rates at consultant level in the Irish health system, although there is limited data to confirm this. At consultant level (the most senior level of hospital doctor), the HSE in 2018 reported that ‘there were… 349 unmatched consultant positions in hospitals and community settings, and that an unmatched position is an indication of a vacancy’ [12]. The Public Appointments Service (PAS) recruit for some, but not all, consultant vacancies (certain types of hospitals can recruit directly). In 2017, PAS advertised 111 consultant posts on behalf of the HSE [12]. The 111 posts advertised in 2017 received, on average, 2.9 applicants each [12], for posts that would have historically attracted a much higher degree of competition. A further indication of the challenging recruitment environment is the situation whereby 127 hospital doctors are working as consultants without being registered on the Specialist Division of the Medical Register (which would generally be considered a pre-requisite for a consultant post) [12].
5. Post-2008, Australia has emerged as the main destination country for emigrant Irish-trained doctors ahead of the more traditional destinations of the United Kingdom and the United States of America [17]. As Ireland does not track the emigration or return of its doctors, the authors cannot say whether this emigration is temporary or permanent. Recent research with emigrant doctors [14, 15] noted that the majority intend to remain overseas. If true of the wider population of emigrant Irish-trained doctors, it poses a risk to the future medical workforce and to the Irish health system.

### Source country drivers of doctor emigration

In addition to the wider recession in Ireland, the Irish health system post-2008 has endured ‘radical resource cuts’ [16], with major reductions in both health spending and health staffing levels [5, 16, 17]. This has placed the Irish health system under considerable strain. Dissatisfaction with deteriorating working conditions [20] has become a driver of emigration as ‘demoralised doctors and nurses are choosing to vote with their feet… and migrate’ [5]. The public sector recruitment embargo 2009–2014 did not apply to doctors [12], but senior hospital doctors (consultants) have been impacted by a number of austerity-related pay cuts. New entrant salaries were reduced by 10% in 2011, in line with other public sector workers [12], and new entrant consultant salaries were reduced by a further 30% in 2012 [12]. This has contributed to the frustration with both salary levels and working conditions at consultant level, a frustration shared by newly appointed consultants and also by eligible candidates who opt to emigrate rather than to apply for these posts.

### Destination country policy levers facilitating doctor emigration

Australia uses two key policy levers which facilitate the migration of doctors to Australia. These policies, the Competent Authority Pathway and the ‘457’ visa programme [21], assist the migration of doctors from Ireland to Australia. The Competent Authority Pathway was introduced by the Australian Medical Board in 2008 and applies to medical graduates of New Zealand, the United Kingdom, Ireland, the United States of America and Canada seeking registration in Australia [21]. It offers a ‘fast track registration’ to Irish-trained doctors seeking to work in Australia, where their medical qualifications are considered equivalent to Australian qualifications. This enables Irish-trained doctors to join the medical register in Australia without further examinations. This ease of registration is a key enabler of doctor migration, as previous research has highlighted [13]. The ‘457’ visa scheme, introduced in 1996, was a temporary visa scheme to bring high skilled workers to Australia [22]. Under the ‘457’ scheme, visas were issued for up to 4 years and could be tied to a specific employer [21, 22]. These visas are popular with health employers seeking to entice doctors to take up posts in areas of need and thus were used as a means of addressing Australia’s medical maldistribution [21]. The ‘457’ scheme was replaced with the ‘482’ visa scheme in 2017/2018.

In quantifying doctor emigration from Ireland to Australia in the decade since 2008, this paper illustrates the impact that an external ‘shocks’, such as economic recession and austerity, can have on the medical workforce. Ireland, like many other source countries, does not quantify emigration or doctor emigration in terms of departure records [7, 17]. In such a situation, data sharing between the source and destination countries can help the source country (in this case, Ireland) to better understand doctor emigration [17] and can inform its policy response. This is why cross-national data sharing is a key recommendation of the WHO Global Code on the International Recruitment of Health Personnel [17, 32]. With another external shock (Brexit) imminent^3^ [18], this paper illustrates how destination country data can help the Irish health system to quantify and respond quickly to emerging patterns of doctor emigration.

## Methods

This study draws on data from three Australian sources to better understand doctor migration flows from Ireland to Australia, post-2008. No ethical review was required for this paper as it used anonymised, pre-collected data which were publically available [33].

### Immigration data

Data on the number of temporary and permanent visas issued to Irish citizen doctors 2005–2018 were obtained, on request, from the Australian Department of Home Affairs. Further data on the number of Irish citizens granted ‘457’ visas, and the numbers granted working holiday visas were obtained from pivot tables on the Home Affairs website in November 2018. The ‘457’ visas were short term (up to 4 years) visas issued to skilled migrants in eligible professions including nurses, doctors, accountants, engineers and carpenters. The visas were tied to a specific employer [21, 22]. The working holiday maker visa scheme facilitates working holidays for people aged 18–30, usually in relatively unskilled employment [7]. Immigration data are collected based on the *citizenship* of the visa recipient, in this case, providing data on the number of Irish citizens who are also doctors and who have obtained visas for Australia. Alongside Irish citizens who have trained (i.e. obtained their basic medical qualification) as doctors in Ireland, these figures will also include Irish citizens who have trained in medicine in another country (e.g. the United Kingdom, EU) and internationally trained doctors who had become naturalised Irish citizens prior to their migration to Australia, although the authors envisage that these will be in the minority.

### Registration data

Data on the number of Irish-trained doctors registered and employed in Australia 2013–2016 were obtained on request from the Australian Institute of Health and Welfare. The data were derived from an annual re-registration survey (response rate 93–97%) aggregated into a National Health Workforce Data Set on Medical Practitioners 2013–2016. Registration data focuses on the training country of the individual doctor. In this case, it records the number of Irish-trained doctors who have registered in Australia. As Irish medical schools train a significant number of international students each year, this number includes international doctors who trained as doctors in Ireland and then migrate to Australia, but excludes Irish citizens who received their medical training in another country (e.g. the United Kingdom, EU) and then migrated to Australia.

### Census data

Census data was obtained, on request, from the Australian Bureau of Statistics. The Census data illustrates how many Irish-born people, reporting their profession as ‘medical professional’, were present in Australia on census night in 2006, 2011 and 2016. Census data include individuals who were born in Ireland, but migrated from Ireland prior to medical training, and exclude those who were born outside of Ireland but obtained their medical training in Ireland.

## Results

### Irish emigration to Australia 2005–2018

The emigration of Irish citizens from Ireland to Australia increased sharply post-2008. The number of ‘457’ visas issued to Irish citizens increased from 3126 in 2008/2009 to a peak of 10 291 in 2012/2013. The number of working holiday visas issued to Irish citizens also increased during the period, from 17 133 in 2007/2008 to 25 827 in 2011/2012 (see Fig. 1). As the Irish economy began to recover from 2014 onwards, the number of ‘457’ visas issued to Irish citizens returned to pre-2008 levels by 2015 (see Fig. 1). The number of working holiday visas issued to Irish citizens also, for the most part, returned to pre-2008 levels by 2015 (the upswing in working holiday visas in 2016/2017 probably related to the anticipated ending of the ‘457’ scheme, which received widespread media coverage in Ireland at the time). The decrease in the number of Australian work visas issued to Irish citizens from 2013 onwards was most likely related to the revival of the Irish economy.

### Irish doctor emigration to Australia 2008–2018

**Fig. 2:**
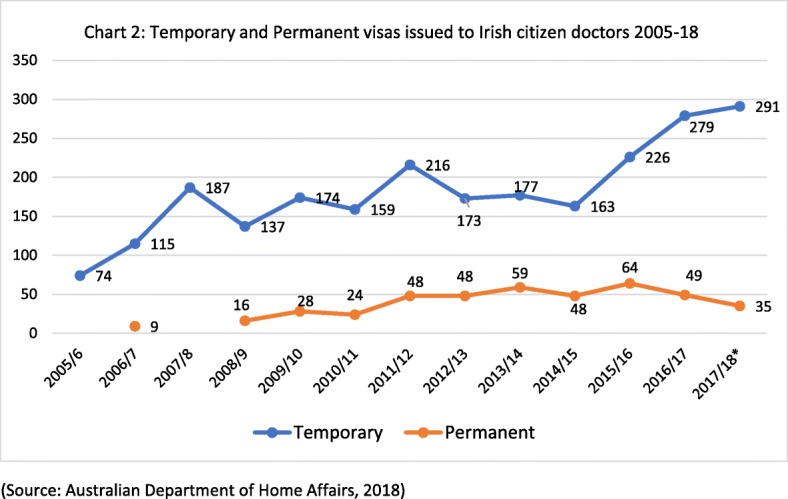
Temporary and permanent visas issued to Irish citizen doctors 2005–2018 (source: Australian Department of Home Affairs, 2018)

The emigration of Irish-trained doctors to Australia is a sub-set of this larger migration from Ireland to Australia post-2008. It might be expected that doctor migration would follow the same patterns, i.e. peaking between 2011 and 2013 before returning to pre-2008 levels by 2014 as the Irish economy showed signs of improvement. However, as Fig. 2 illustrates, the number of Irish citizen doctors granted ‘457’ visas increased in the period 2008–2012 and has continued to increase. In 2017/2018, a decade since the onset of recession in Ireland, 326 Irish citizen doctors were issued with working visas (temporary and permanent) for Australia, more than double the 153 issued in 2008/2009. This trend suggests that the migration of doctors is not primarily related to economic circumstances, which began to recover in 2013/2014, but perhaps to health system factors.

**Fig. 3:**
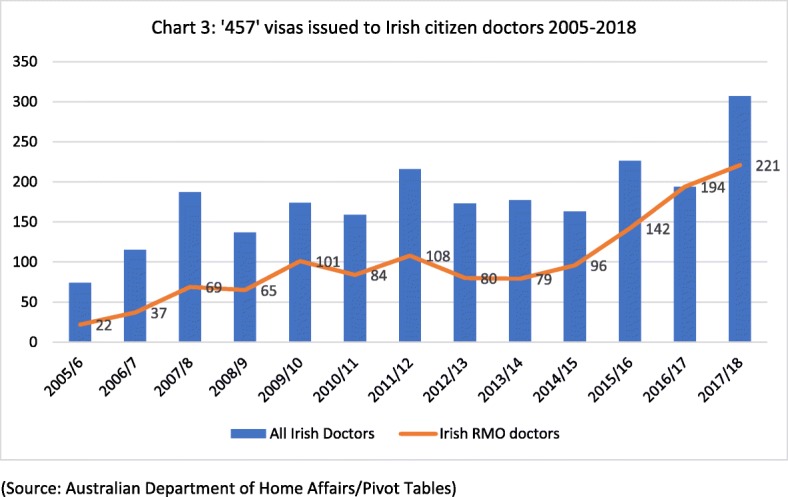
‘457’ visas issued to Irish citizen doctors 2005–2018 (source: Australian Department of Home Affairs/Pivot Tables)

Figure 3 presents data on the number of Irish citizen doctors obtaining ‘457’ visas to take up RMO/resident medical officer posts in the Australian health system. These posts are typically occupied by early career doctors who have recently completed their medical degree and internship in the Irish health system and are either pre-training, or in the early stages of postgraduate medical training. The number of doctors migrating from Ireland to Australia at this early career stage increased from 22 in 2005/2006 to 221 in 2017/2018. In 2017/2018, 221 of the Irish doctors granted 457 visas were early career stage doctors, while the remaining 86 were more senior doctors.

### A profile of Irish-trained and Irish-born doctors in Australia

**Fig. 4:**
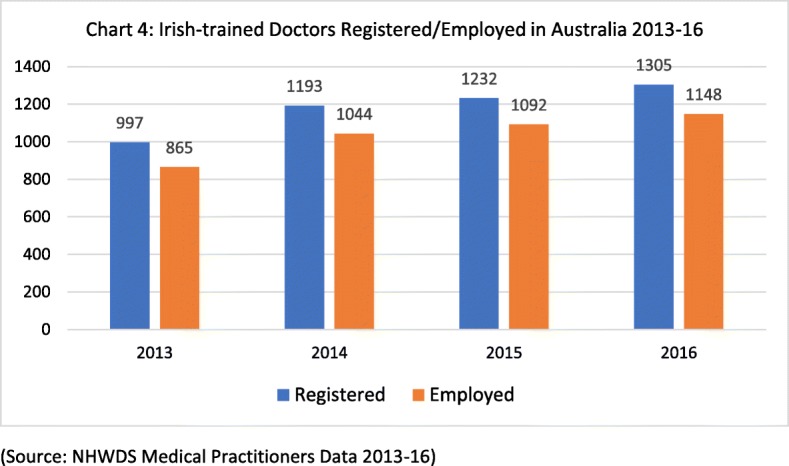
Irish-trained doctors registered/employed in Australia 2013–2016 (source: NHWDS Medical Practitioners Data 2013-16)

Figure 4 draws on employment and registration data to indicate the ‘stock’ of Irish-trained doctors in Australia 2013–2016. The number of Irish-trained doctors registered in Australia increased from 997 in 2013 to 1305 in 2016. As it is clear from Fig. 4, there is a slight difference (roughly 10%) between the numbers registered and the numbers employed.

**Fig. 5:**
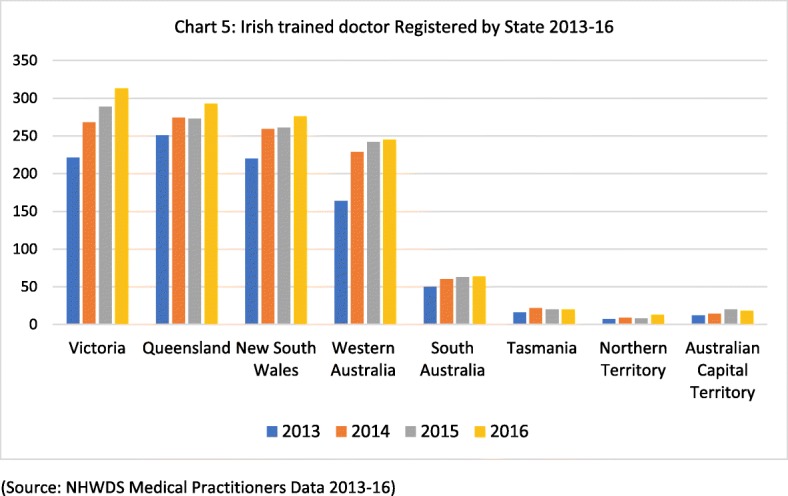
Irish-trained doctor registered by state 2013–2016 (source: NHWDS Medical Practitioners Data 2013-16)

**Fig. 6:**
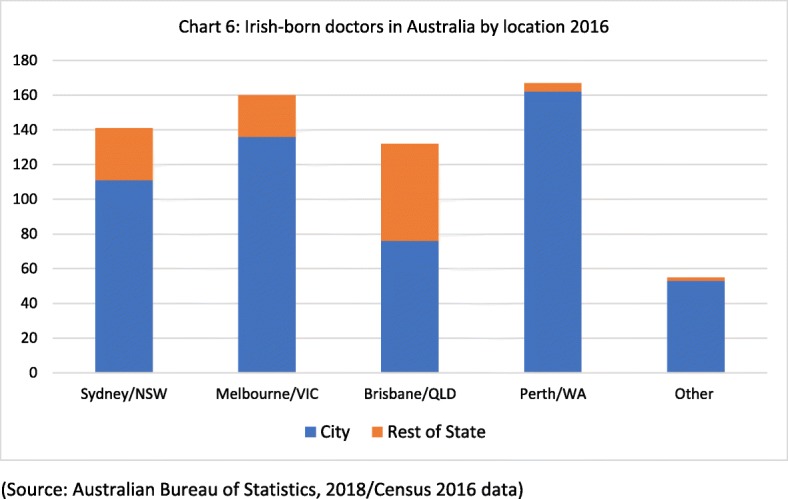
Irish-born doctors in Australia by location 2016 (source: Australian Bureau of Statistics, 2018/Census 2016 data)

Of the Irish-trained doctors registered in Australia 2013–2016, the majority are based in Victoria, Queensland, New South Wales or Western Australia (see Fig. 5), rather than in the smaller states and territories. This is also borne out in the 2016 Census data (Fig. 6), which shows that Irish-born doctors in Australia are predominantly based in the major urban centres of Australia—Sydney, Melbourne, Brisbane and Perth. That is both typical of skilled migration to Australia and an indication of the challenge of attracting skilled health workers to regional areas of Australia.

Data from the Australian Census also show an increase in the ‘stock’ of Irish-born doctors resident in Australia from 2006 to 2016, from 321 in 2006, and 460 in 2011 to 655 in the most recent Census of 2016. NHWDS data also shows the main grades held by Irish-trained doctors registered in Australia 2013–2016. It shows that within the stock of 1305 Irish-trained doctors registered in Australia in 2016, 402 were registered as specialists, 375 as general practitioners, 234 as specialists in training, 132 were hospital non-specialists and 162 were categorised as ‘other’ (other clinicians, non-clinicians or non-applicable).

## Discussion

### Austerity and push-pull

The 2008 recession and subsequent austerity period was a significant driver of emigration from Ireland. Australia was a key destination for these post-2008 Irish emigrants. This emigration flow from Ireland to Australia began to slow in line with Ireland’s economic recovery (from 2014 onwards) and, as of 2017/2018, had returned to pre-2008 levels. At a glance, this wider population migration pattern appears to be in line with the push-pull model [19] as workers were ‘pushed’ from austerity-constrained Ireland and ‘pulled’ by the availability of better job and quality of life prospects in Australia. However, doctor emigration from Ireland to Australia has not followed the same pattern. Although it initially increased post-2008 in line with the economic downturn, the rate of doctor emigration has not reduced in line with Ireland’s economic recovery. This indicates that doctor emigration is driven by factors other than national economic circumstances and push/pull factors.

When modified to incorporate health system factors, the push-pull model can offer useful insights, whereby doctors are pushed to emigrate from Ireland due to ‘push’ factors within the Irish health system (rather than in the wider economy), such as austerity-related health budget cuts [16], deteriorating working conditions [20] and reductions in new entrant salaries for senior hospital doctors (consultants). The decision to migrate is also influenced by ‘pull’ factors in Australia. A significant attraction of Australia is its reputation among Irish-trained doctors and nurses for providing good working conditions for health workers and good staffing levels [20]. A comparison of senior staffing levels reveals that Australia has 6.8 emergency medicine consultants per 100 000 population, whereas Ireland has 2.2 emergency medicine consultants per 100 000 population. Research with Irish-trained emigrant doctors in 2015 [34, 23] bears this out, highlighting the challenges faced by doctors working within an ‘austerity-constrained health system’ [34] and the fact that the decision to opt for emigration tended to be informed by professional (rather than personal) factors [20] and the generational desire for a better balance between work and life [23].

### Beyond push-pull

In understanding doctor migration from Ireland to Australia, the push-pull model provides an important starting point; however, there are other considerations which help to explain why doctor emigration has continued despite Ireland’s recent economic recovery.

The first was discussed by Glynn et al. who applied the concept of social resilience in relation to Irish emigration, post-2008 [2]. Social resilience is defined as the ‘capacity of groups of people bound together… to sustain and advance their well-being in the face of challenges to it’ [24]. In countries worst-hit by the 2008 economic crisis, some individuals used emigration in order to sustain their ‘pre-crisis wellbeing’ [2]. This option was available only to those who were mobile and who had the skills, qualifications and language skills required by the destination country. This relates to the ‘structural context within which’ [19] the migration can occur, i.e. the extent to which the destination country is open to migration. Irish-trained doctors, as highly skilled, English-speaking migrants, have been able to access ‘fast track’ processes which have enabled them to obtain registration, visas and employment in Australia with relative ease.

Another factor in facilitating the migration of Irish-trained doctors from Ireland to Australia, relates to the medical profession. Professional networks (earlier cohorts of Irish-trained doctors) help Irish-trained doctors to access employment opportunities in Australia. These networks ‘facilitate the likelihood of international movement because they provide information which lowers the costs and risks of migration’ [25] and in this way, they help to ensure the continuation of the pattern of migration [19, 26]. This constitutes a typical form of chain migration.

The Irish medical profession also facilitates and encourages doctor migration via a culture of medical migration [17]. This culture of migration presents migration to medical students and early career doctors, ‘as an essential (rather than an optional) component of a successful medical career’ [17]. This culture of medical migration evolved with the expectation of circular or return migration, i.e. that emigrant doctors would return to work in the Irish health system with the skills obtained abroad; however, this return migration cannot be presumed.

The dynamics of doctor migration from Ireland to Australia are an important reminder of the complexity of doctor migration flows and the inter-connectedness of the source and destination countries and health systems. External shocks to the Irish health system, such as recession and austerity, provided the initial trigger for the initial migration of doctors from Ireland to Australia, post-2008,, but further waves of emigrant doctors were sustained by the success of the earlier cohorts Irish-trained doctors in Australia and by a professional and national culture of emigration, At a collective level, this has helped to ensure the perpetuation [19, 26] of the migration flow of doctors from Ireland to Australia post-2008.

The key question is whether and for how long this migration pattern will continue. With another external shock (Brexit) on the horizon [18], it is important for the Irish health system to carefully consider the impact of external shocks on doctor emigration patterns, as well as considering the impact of doctor emigration on the Irish health system.

### Implications of doctor emigration on the Irish health system

While doctor emigration can be positive for the health system, the scale of doctor emigration from Ireland in recent years means that the negative implications, namely ‘medical brain drain, a high dependence on internationally trained doctors and a highly transient medical workforce’ [17], must also be considered. The cost or benefit of doctor emigration to a source country, such as Ireland, depends on whether or not emigrant doctors return. Circular or return migration for the acquisition of sub-specialist skills has benefitted the Irish health system in the past [17]. However, there is no guarantee of return and post-2008 doctor emigration may differ from previous waves of doctor emigration. Recent research with emigrant Irish-trained doctors indicates that the majority intends to remain overseas [14, 15].

In 2014, Ireland graduated 684 EU/Irish doctors^1^ [27] and in the same year, 627 doctors emigrated to Australia, the United Kingdom, the United States of America, Canada and New Zealand [17]. Ireland is losing a large number of doctors relative to the number of doctors it trains and therein lies the problem. Doctor emigration on a large scale represents a financial loss to the Irish health system. The financial cost of training, but then losing, medical graduates is relatively straightforward to calculate and has been estimated at between €105 000–€126 000 per doctor trained via undergraduate medicine [28]. However, large-scale doctor emigration also means the loss of talent, experience and potential from the Irish health system. This is far more difficult to quantify, although important to remember. As Kapur explains, ‘any system that haemorrhages talent over the long run will struggle to survive let alone prosper’ [29] and these risks to the Irish health system have yet to be acknowledged.

As Ireland’s rate of doctor emigration has increased post-2008, its reliance on internationally trained doctors to staff the health system has also increased from 13.4% in 2000, to 33.4% in 2010 [9] to 42% in 2017 [11] (the overall medical workforce increased by 29% during this timeframe, from 7115 in 2007 to 9193 in 2017 [12]). Irish doctors migrate to Australia to work in the Australian health system, while doctors from Sudan, India and Pakistan (countries which themselves have shortages of doctors) migrate to work in the Irish health system. Replacing emigrant Irish-trained doctors with internationally trained doctors has enabled the Irish health system to change the doctors in the system rather than changing the conditions with which they were dissatisfied [30]. Internationally trained doctors are more likely to fill the least desirable posts in the Irish health system—those with minimal opportunities for postgraduate specialist training or career progression, for instance 77% of Ireland’s non-training posts are occupied by internationally trained doctors [12]. Unsurprisingly, the mismatch between expectations and reality for internationally trained doctors in Ireland causes dissatisfaction [31] and encourages their onward migration to other destination countries. Replacing Irish-trained doctors with internationally trained doctors and then replacing those internationally trained doctors who emigrate ensures that Ireland has a ‘highly transient medical workforce’ [23]. Encouraging a more stable workforce will require addressing the underlying reasons for Ireland’s high rate of doctor emigration.

### Dynamics of doctor migration and value of data sharing

There is a need to generate quantitative and qualitative data on emigration and return to inform nuanced and up-to-date debate on doctor emigration and in order to fully comprehend the implications of doctor emigration for the Irish health system.

As this paper has demonstrated, Ireland should not assume that historic patterns of emigration and return will set the scene for future emigration trends. The emergence of Australia as the key destination of Irish-trained doctors in the decade since 2008 is a timely reminder of the need for up-to-date information on doctor emigration. Despite (or perhaps because of) its long history of emigration, Ireland does not generate data on departures [7] and does not collate data on doctor emigration, or track their return. Ireland should begin to generate this data, cross-referencing it with entry data (working visa, registration) from key destination countries, to better understand and respond to emerging doctor emigration trends. An evidence-based policy response to doctor emigration is required.

## Conclusion

The data on the migration of Irish doctors to Australia indicate a very high level of migration to Australia since 2008—to the extent that Australia is now the main destination for Irish-trained doctors. The outward flow of doctors from Ireland has both been a function of economic circumstances in Ireland 2008–2018, but more especially of salaries and working conditions in the Irish health system, and of continued economic growth in Australia, that has made it an attractive destination. That has in turn resulted in a high level of replacement migration economic costs as Irish-trained doctors are replaced by internationally trained doctors. The resulting high level of transience or ‘churn’ in the Irish health system may be reducing the effectiveness of health care delivery in Ireland.

Addressing the retention crisis facing the Irish health system must begin with better data. Alongside data which will ‘track’ doctors exit and return to the Irish health system, better workforce data is also required (e.g. on the number of vacant posts in the workforce and their specialty/location, demographic information on the medical workforce in terms of grade, gender, age, nationality). This will enable policy makers to remain abreast of emerging emigration and workforce trends and in a position to respond by using appropriate policy levers. However, improved retention will also require an understanding of why doctors emigrate and what might encourage or discourage them from return. A follow-up paper presenting qualitative data from interviews with Irish-trained doctors in Australia conducted in 2018 will explore these issues in greater detail [15]. Understanding how many doctors are emigrating, where they are leaving to and why, is vital to the future health of the Irish medical workforce and to the wider health system.

## Abbreviations

EM: Emergency medicine
EU: European Union
NHWDS: National Health Workforce Data Set
NSW: New South Wales
QLD: Queensland
RMO: Resident Medical Officer
UK: United Kingdom
USA: United States of America
VIC: Victoria
WA: Western Australia
WHO: World Health Organization

## Footnotes

1 When referring to Irish-trained doctors or doctors trained in Ireland within this paper, the authors refer to doctors who are Irish/EU passport holders who obtained their basic medical qualification in Ireland. Ireland also trains a significant number of doctors from non-EU countries, but the presumption has been that these doctors do not remain on to work in the Irish health system. In fact they face barriers (e.g. access to internship) if attempting to remain to practice medicine in Ireland following graduation.

2 The HSE provided nationality data for a sample of its medical workforce, which indicated that 84% of its consultants and just over 50% of its junior hospital doctors in 2017 were Irish 12. Public Service Pay Commission, *Report of the Public Service Pay Commission. Recruitment and Retention Module 1*. 2018, Public Service Pay Commission Dublin.

3 Brexit may see Irish-trained doctors returning from the UK to Ireland, opting to migrate to Australia, rather than to the UK; or perhaps the UK will actively recruit Irish trained doctors, if the UK and Ireland retain a Common Travel Area post-BREXIT.
